# Mycobacterium Kansasii Infection and Microscopic Polyangiitis: An Unexpected Association

**DOI:** 10.7759/cureus.2831

**Published:** 2018-06-18

**Authors:** Ammar Alnaser, Konstantinos Parperis

**Affiliations:** 1 Internal Medicine/Nephrology, UCLA Medical Center, Los angeles, USA; 2 Medicine, University of Arizona College of Medicine/mihs, Phoenix, USA

**Keywords:** mycobacterium kansasii, microscopic polyangiitis, crescentic glomerulonephritis, myeloperoxidase antibody

## Abstract

*Mycobacterium kansasii* (*M. kansasii*) is a nontuberculous mycobacterium, which mainly infects the lungs in immunocompromised patients. We present here the case of a 27-year-old immunocompetent patient who developed pulmonary-renal syndrome, manifested with lung cavitation, miliary nodules, and necrotizing glomerulonephritis accompanied by elevated titers of myeloperoxidase antibody. Cultures from bronchoalveolar lavage were positive for *M. kansasii*, and the patient was treated with an anti-mycobacterial regimen. Additionally, given the presence of the myeloperoxidase antibody and glomerulonephritis on renal biopsy, our patient was diagnosed with microscopic polyangiitis (MPA). Unfortunately, the patient stopped his regimen and developed worsening respiratory failure and died. To our knowledge, this is the first case associating an *M. kansasii* infection with MPA, although more studies are needed to confirm this finding.

## Introduction

*Mycobacterium kansasii* (*M. kansasii* ) is an acid-fast bacillus that belongs to the mycobacterium family, and the most common manifestation is chronic pneumonia with fever, cough, and hemoptysis [1]. In addition, patients may develop weight loss, cavitary lung lesion, lymphadenitis, and disseminated disease [2]. Microscopic polyangiitis (MPA) is an autoimmune disease characterized by systemic, pauci-immune necrotizing, small vessel vasculitis and the presence of perinuclear-anti-neutrophil cytoplasmic antibodies (p-ANCA), directed against the myeloperoxidase [3]. The disease may present as a pulmonary-renal syndrome, with alveolar hemorrhage, respiratory failure, and rapidly progressive glomerulonephritis. The mechanism of the MPA remains unknown, but studies implicated the role of anti-myeloperoxidase antibody in the pathogenesis of the disease [4]. 

We present here the case report of a male patient who presented with pulmonary-renal syndrome and was found to have *M. kansasii* and MPA with positive myeloperoxidase antibody. Concurrent *M. kansasii* infection and MPA disease have been never before reported in the literature.

## Case presentation

A 27-year-old man was admitted to our hospital with a two-week history of hemoptysis, shortness of breath, and fever. He reported fatigue, night sweats, bilateral knee pain, and a rash on his back and chest. Two months prior to this admission, the patient developed acute onset of nonproductive cough associated with generalized weakness and muscle pain. For his musculoskeletal symptoms, the patient was prescribed prednisone 20 mg daily for five days by his primary care physician without improvement in his symptoms.

**Figure 1:**
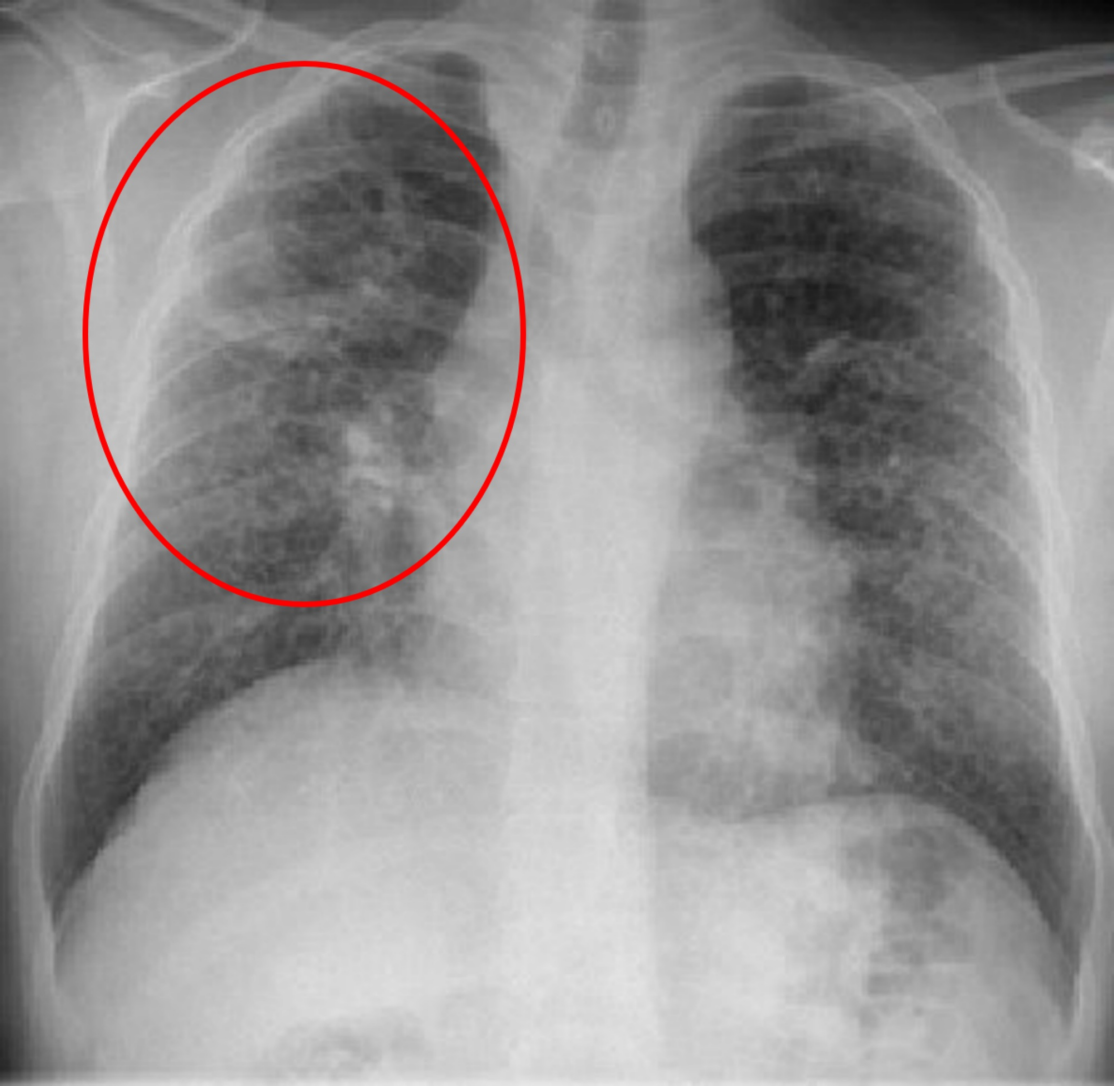
Chest radiograph showing right upper lobe cavitary lung lesion and diffuse nodular reticular interstitial opacities.

At the time of admission, his blood pressure was 130/69 mmHg, heart rate was 99 bpm, temperature was 37.8°C (100°F), and respiratory rate was 17 breaths per minute. He was found to have bilateral diffuse rales on chest auscultation and a morbilliform rash on his back and upper chest. Laboratory studies showed his white blood cell count was 13.2 × 103/ml, erythrocyte sedimentation rate was 122 mm/hour (reference rate, <20 mm/hour), hemoglobin was 10.4 g/dl, serum creatinine was 1.48 mg/dl (reference range, 0.6–1.1 mg/dl), and glomerular filtration rate was 57 ml/min/1.73 m2 (reference rate, > 90 ml/min). Urinalysis showed proteinuria and hematuria with few red blood cell casts. A radiograph of the chest demonstrated diffuse nodular reticular interstitial opacities and right upper lobe cavitation (Figure 1).

**Figure 2:**
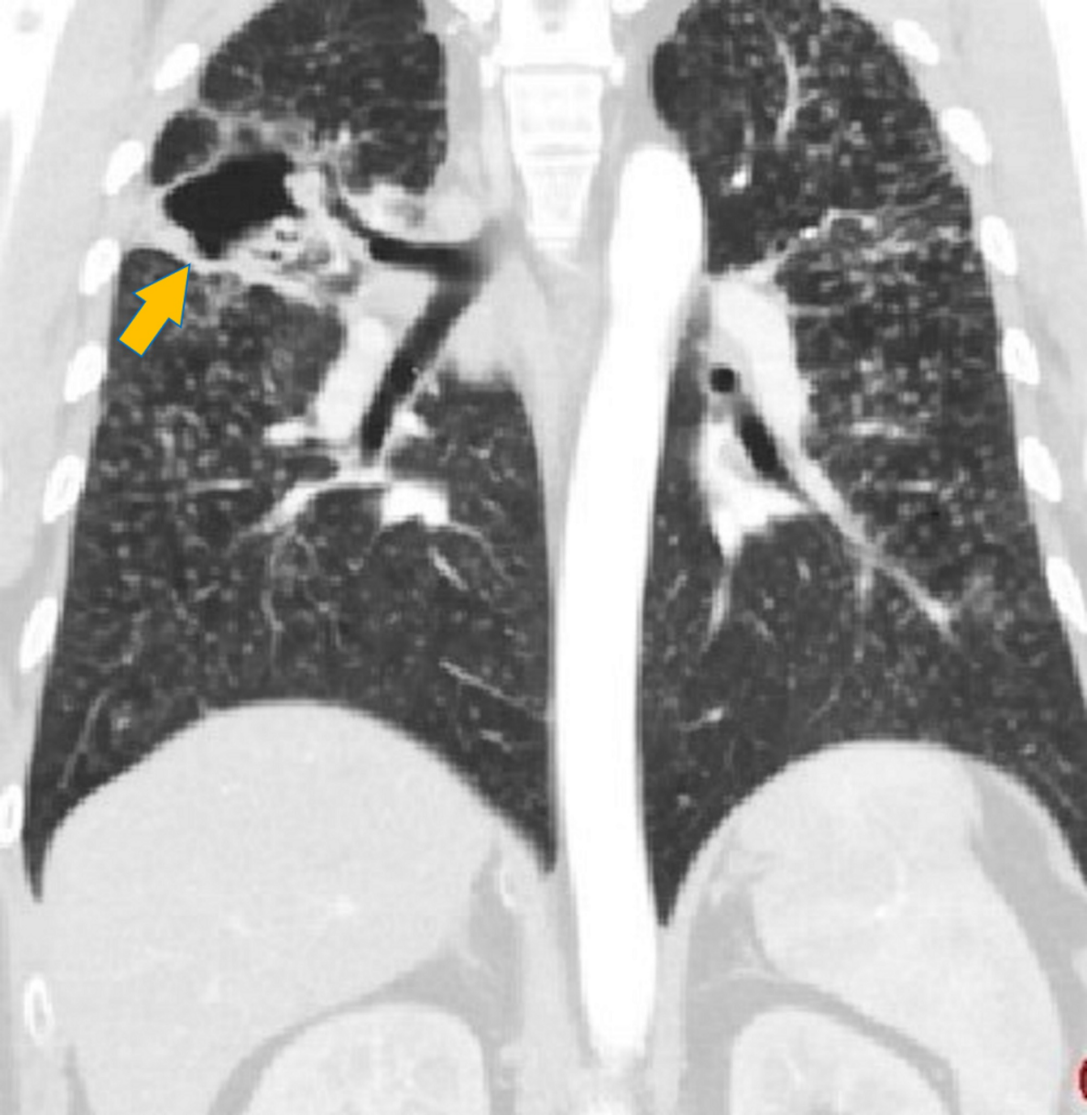
Computed tomography scan of the chest revealing a 4.7-cm right upper lobe cavitary lesion and multiple bilateral nodules in a miliary pattern throughout the lungs with mediastinal lymphadenopathy.

The patient was admitted to the intensive care unit with respiratory failure, and he was intubated. A computed tomography scan of the chest revealed a 4.7-cm right upper lobe cavitary lesion and multiple bilateral nodules in a miliary pattern throughout the lungs with mediastinal lymphadenopathy (Figure 2).

**Figure 3:**
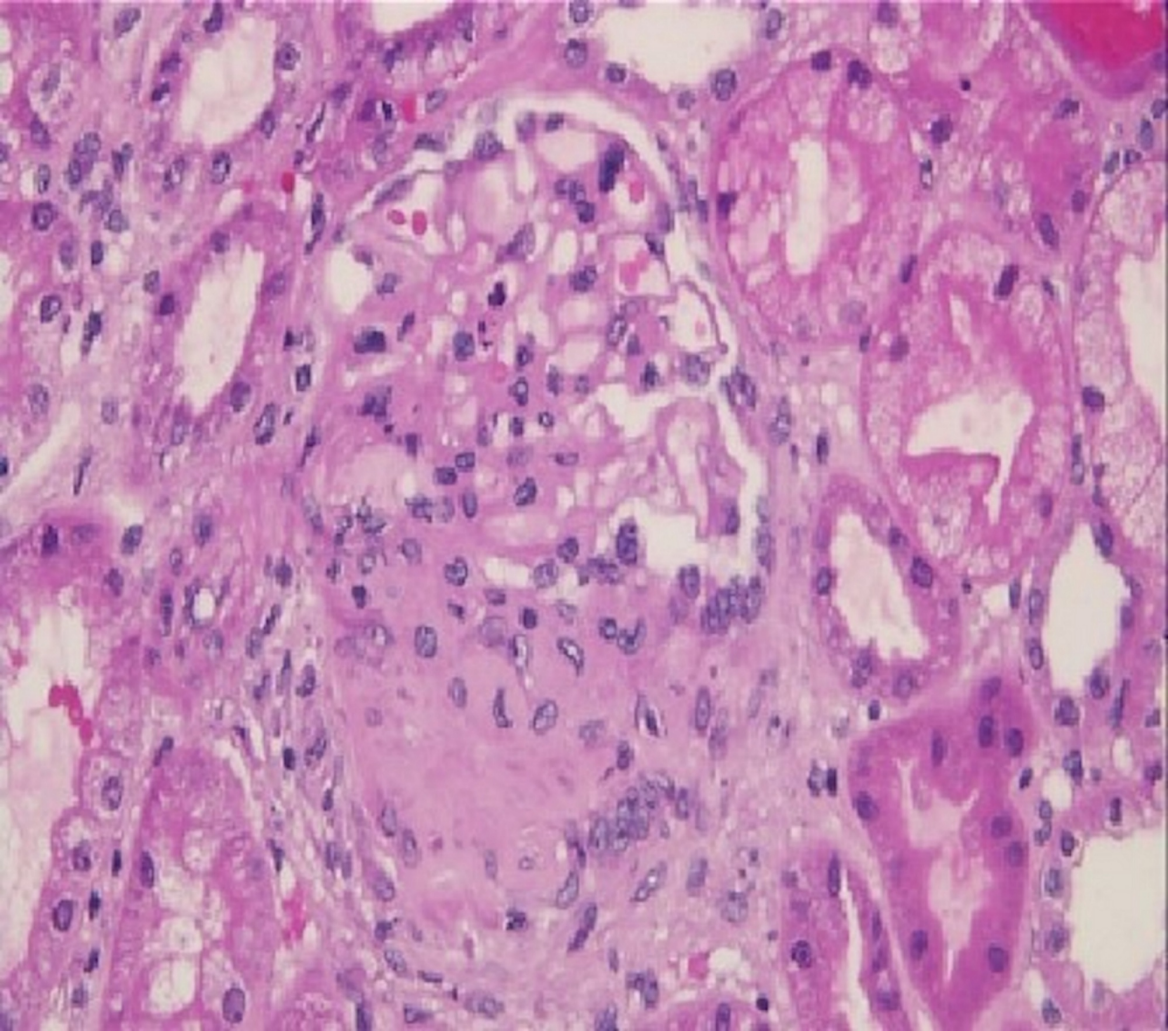
Renal biopsy revealed crescentic and necrotizing glomerulonephritis.

**Figure 4:**
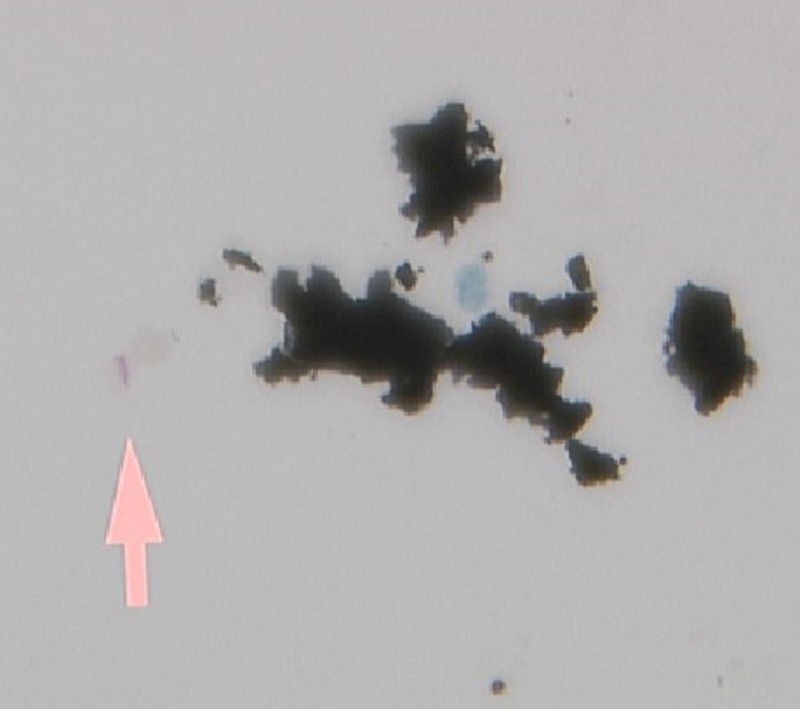
Acid-fast stain positive in sputum.

Given the clinical features and imaging findings, we considered an infection with mycobacterium tuberculosis as the main cause of his symptoms. Therefore, empirical treatment with rifampin (600 mg oral daily), ethambutol (1200 mg oral daily), isoniazid (300 mg oral daily), and pyridoxine (50 mg daily) was initiated. The patient underwent bronchoscopy, the findings of which were unremarkable and acid-fast bacilli (AFB) smear and cultures were sent. In addition, pulmonary-renal syndrome was included in our differential diagnosis based on hemoptysis, hematuria, and proteinuria. Thus, immunologic studies were obtained, and the p-ANCA was positive, with a titer of 1:160 (negative, < 1:20) as well as the anti-myeloperoxidase (MPO) antibody with a titer of 150 U/mL (negative, < 9). Antinuclear antibody, proteinase-3 antibody, and glomerular basement protein antibody were not detected. The results of the QuantiFERON-TB gold test was indeterminate, human immunodeficiency virus (HIV) antigen and antibody test results were negative, and coccidioidomycosis antibodies IgM and IgG test results were normal. Further evaluation with renal biopsy demonstrated crescentic and necrotizing glomerulonephritis (Figure 3) with mesangial immune complex deposits and 25% tubulointerstitial fibrosis. Given the hemoptysis, cavitary lung lesion, renal failure due to necrotizing glomerulonephritis, elevated anti-MPO antibody levels, the absence of upper respiratory system symptoms, and granulomas on renal biopsy, the patient was diagnosed with MPA and treatment with prednisone 60 mg daily was initiated. Due to concerns for an active mycobacterial infection, the patient was not treated with intravenous (IV) corticosteroids and/or immunosuppressive agents like rituximab or cyclophosphamide. His symptoms were slowly improving, and the patient was extubated. Two weeks later, multiple sputum AFB stains (Figure 4) and cultures were found to be positive for *M. kansasii*, which confirmed our suspicion of mycobacterial infection, and we continued the anti-mycobacterial regimen. Considering a serious lethal infectious process and the patient’s clinical improvement, the prednisone was tapered to 30 mg daily, and additional immunosuppressive treatment was deferred until the patient received at least eight weeks of consolidative antimycobacterial therapy. The patient’s symptoms continued to improve, and he was discharged after four weeks of hospitalization.

 

Unfortunately, the patient stopped the anti-mycobacterial regimen and prednisone two weeks after he was discharged due to nausea and vomiting. One week later, he experienced recurrent episodes of hemoptysis and shortness of breath, and he was readmitted to the hospital where he was intubated due to acute hypoxic respiratory failure and diffuse alveolar hemorrhage. Renal function and acidosis worsened, and continuous renal replacement therapy was initiated. The working diagnosis was active MPA with coexistent *M. kansasii* infection. He was started on an anti-mycobacterial treatment, plasmapheresis, IV methylprednisolone 1000 mg daily for three days and then to 60 mg daily, and IV cyclophosphamide 850 mg was given once. Unfortunately, the patient developed shock and multi-organ failure; he was placed on multiple vasopressors and antibiotics without response, and seven days after this admission, he died.

## Discussion

Mycobacterium is a genus of Gram-positive bacilli that demonstrates the property of acid-fastness. *M. kansasii* is a nontuberculous mycobacterium, one of the more virulent pathogens in this genus that can infect immunocompromised and, less often, immunocompetent patients. The highest incidence of *M. kansasii* infection occurs in the Southwest and Midwest regions of the USA [1]. It is considered the second most common nontuberculous mycobacteria pathogen in the USA, preceded by mycobacterium avium complex [2]. Infection might occur sporadically from environmental sources (primarily tap water); no evidence yet suggests person-to-person transmission [2]. Although most cases include middle-age Caucasian patients, the infection can affect any age, sex, and ethnicity. Patients with pre-existing pulmonary conditions like chronic obstructive pulmonary disease, pneumoconiosis (e.g., silicosis), and alcoholism have a higher risk of infection. Clinical features are indistinguishable from tuberculosis and include constitutional symptoms (e.g., fever, weight loss, anorexia), papular rash, cervical lymphadenitis, osteomyelitis, meningitis, pneumonia, or disseminated disease. The lungs are the most commonly affected organ, and patients may present with chronic productive cough, hemoptysis, shortness of breath, and dyspnea. In addition, patients with an HIV infection may develop disseminated disease that can be fatal. Diagnosis is based on the identification of the mycobacterium from sputum, tissue, or blood cultures. Histologic findings include caseating and noncaseating granulomatous inflammation. The management of an *M. kansasii* infection includes antituberculous drugs like rifampin, isoniazid, ethambutol, streptomycin, and clarithromycin [2]. The ANCA-associated vasculitis is a group of systemic autoimmune diseases, and that includes granulomatosis with polyangiitis, MPA, and eosinophilic granulomatosis with polyangiitis. MPA is characterized by necrotizing small vessel vasculitis and the presence of p-ANCA, directed against the myeloperoxidase [3]. The disorder manifests with myriad symptoms and may affect multiple organs including the kidneys, lungs, nerves, skin, and joints. The main pathologic features of MPA include pulmonary capillaritis, necrotizing crescentic glomerulonephritis with absent or minimal deposition of immunoglobulins and complement, without evidence of granulomas. Although the pathogenesis has not been clarified, animal studies implicate the role of anti-MPO antibodies in the pathophysiology of the disease via neutrophil activation [4]. Radiographic findings may resemble a mycobacterial infection and include bilateral nodular, patchy opacities, and, less frequently, cavitary lung lesions. 

For several decades, infections have been considered as a possible cause or trigger of systemic autoimmune disease, but this topic remains controversial. In the present case, we believe the mycobacterial infection in the lungs induced an immunologic response that led to the production of anti-MPO antibody, resulting in necrotizing glomerulonephritis and possible alveolitis. A possible link has been flagged in several reports evaluating the role of heat shock proteins (HSP) in patients with autoimmune diseases. HSPs have a protective role in the cellular environment against ischemic insults, oxidative stress, and infections [5]. Previous studies have proposed that mycobacterial infections can induce the production of these antibodies as cross-reacting antibodies through molecular mimicry and result in autoimmunity [6-7]. A recent study examined the sera of patients with MPA, rheumatoid arthritis (RA), systemic lupus erythematosus (SLE), and healthy controls for anti-mycobacterium HSP 70 antibody and showed that 12% of the patients with MPA were positive compared with 19.1% of the patients in the RA group, none in the SLE group, and 2.5% of the patients in the healthy control group [8]. This suggests a possible role of these antibodies in autoimmune diseases [8]. Furthermore, one paper found elevated levels of the antibody against the mycobacterial HSP 65 in patients with MPO-ANCA and anti-proteinase 3-ANCA vasculitis, compared to healthy controls [9].

Nevertheless, a second possible pathophysiologic explanation for our case is based on Hickam’s dictum, which states that the patient’s symptoms may be due to two unrelated disease processes: infectious and autoimmune. Although it is very rare to see an uncommon autoimmune disease coexisting with a rare infection, it may raise the possibility for an underlying immunodeficiency related to this patient’s phenotype. However, a thorough workup for immunodeficiencies was not obtained.

The *M. kansasii* identification could be due to contamination or colonization, although this explanation is less likely given the radiographic findings and clinical symptoms consistent with mycobacterial infection.

Interestingly, in the present case, a renal biopsy revealed classic histologic features of ANCA vasculitis but with strong immunofluorescence findings uncommon in MPA, pointing to possible post-infectious glomerulonephritis. An *M. kansasii* infection has been not associated with kidney infection or glomerulonephritis.

## Conclusions

This case highlights the possibility of an *M. kansasii* infection triggered autoimmunity leading to MPO-associated vasculitis, manifested with respiratory failure and necrotizing glomerulonephritis. To the best of our knowledge, this is the first report supporting an association of *M. kansasii* infection and vasculitis. More studies are needed to explore the possible connection between vasculitis and infections.
